# Strong polarization switching with low-energy loss in hydrogen-bonded organic antiferroelectrics†
†Electronic supplementary information (ESI) available. See DOI: 10.1039/c7sc03859c


**DOI:** 10.1039/c7sc03859c

**Published:** 2017-11-01

**Authors:** S. Horiuchi, R. Kumai, S. Ishibashi

**Affiliations:** a Flexible Electronics Research Center (FLEC) , National Institute of Advanced Industrial Science and Technology (AIST) , Tsukuba , Ibaraki 305-8565 , Japan . Email: s-horiuchi@aist.go.jp; b Condensed Matter Research Center (CMRC) and Photon Factory , Institute of Materials Structure Science , High Energy Accelerator Research Organization (KEK) , Tsukuba , Ibaraki 305-0801 , Japan; c Research Center for Computational Design of Advanced Functional Materials (CD-FMat) , National Institute of Advanced Industrial Science and Technology (AIST) , Tsukuba , Ibaraki 305-8568 , Japan

## Abstract

The electric-field-induced phase transition from antipolar to polar structures is at the heart of antiferroelectricity. We demonstrate direct evidence of antiferroelectricity by applying a strong electric field to two antipolar crystals of squaric acid (SQA) and 5,5′-dimethyl-2,2′-bipyridinium chloranilate. The field-induced polarization of SQA is quite large and reasonably explained by the theoretically calculated polarization on the hydrogen-bonded sheet sublattice. The pseudo-tetragonal lattice of SQA permits unique switching topologies that produce two different ferroelectric phases of low and high polarizations. By tilting the applied field direction, the electrical switching mechanism can be attributed to a 90° rotation of the sheet polarization. From the viewpoint of applications, the strong polarization, high switching field, and quite slim hysteresis observed in the polarization *versus* electric field curve for SQA are advantageous for excellent-efficiency energy storage devices.

## Abstract 2

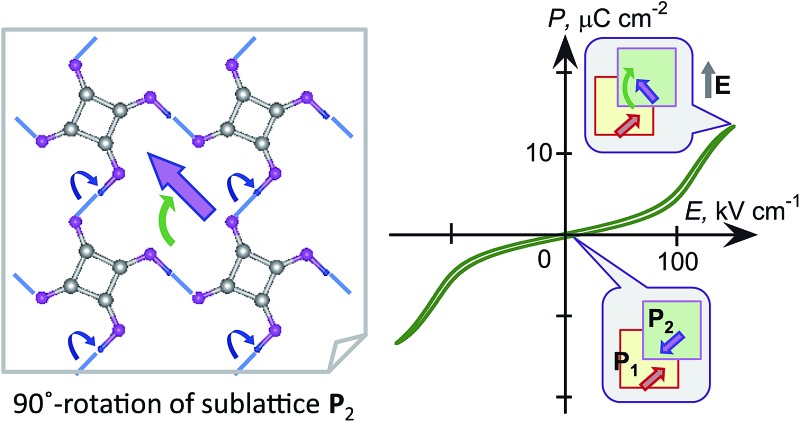
Highly efficient switching of strong polarization in squaric acid crystal arises from 90° rotation of sheet polarization in a pseudo-tetragonal lattice.

## Introduction

In a polar substance or ferroelectric, an external electric field can reversibly switch the direction of spontaneous polarization and induce mechanical strain in the substance through piezoelectricity.^1^,^2^ In the last decade, there have been many intriguing discoveries of strong polarization, low-field switching, and above-room-temperature ferroelectricity in small-molecule organic compounds.^3^,^4^ For many of these small-molecule ferroelectrics, the polarization switching is triggered by proton motion within the intra- and/or intermolecular hydrogen bonds. One of the mechanisms is cooperative prototropy (or “proton tautomerism”), which relocates a hydrogen position and simultaneously interchanges the locations of a single bond and an adjacent double bond. Ferroelectric prototropy has been found in single-component compounds such as croconic acid (CRCA), 2-phenylmalondialdehyde, benzimidazoles, and trisubstituted haloimidazoles.^5^–^8^ This π-bond switching is found to induce a switchable polarization that is larger than that due to proton motion alone and is responsible for the strong spontaneous polarization being comparable to or higher than those of ferroelectric polymers (∼8 μC cm^–2^).^9^ For CRCA, the spontaneous polarization of ∼30 μC cm^–2^ at room temperature is the highest level among organic systems and even exceeds that of BaTiO_3_ (26 μC cm^–2^).^7^ Another class concerns binary molecular compounds, which undergo polarization reversal through the cooperative motion of protons between acid and base molecules.^3^,^10^ Examples are found in neutral or proton-transferred ionic cocrystals of a series of anilic acids (2,5-dihalo-*p*-benzoquinones), in which alternating acid and base molecules with hydrogen bonds construct a ribbon-like supramolecular structure. Through our discovery of more than 15 hydrogen-bonded ferroelectrics, the coercive field typically ranging from 0.5 to 50 kV cm^–1^ turns out to be promising for low-voltage operations in comparison with those of ferroelectric polymers exceeding several hundreds of kilovolts per centimeter. The ferroelectric phase is often thermally robust so that the Curie point *T*_c_ is even hidden beyond the thermal stability limit of the substances themselves (temperatures of fusion, sublimation, or decomposition).

Besides ferroelectrics, there are another fascinating class of dielectrics having switchable electric polarizations. Antiferroelectrics undergo a reversible switching of the electric dipoles from antipolar to polar arrangements and *vice versa* with the application and removal of an electric field, respectively.^1^,^2^ The antipolar crystal structure is closely comparable in terms of free energy to its ferroelectric modification so that the electric polarization (*P*) *versus* electric field (*E*) hysteresis of a ferroelectric is interrupted at an additional *P* ∼ 0 state near the zero-field bias. Since the theoretical prediction by Kittel^11^ followed by experimental confirmation in PbZrO_3_ ceramics^12^,^13^ in the early 1950s, antiferroelectricity has been revealed mainly in oxides and inorganic salts as exemplified by many lead perovskite compounds, NaNbO_3_, NH_4_H_2_PO_4_, and Hf_1–*x*_Zr_*x*_O_2_, but it has received much less interest than ferroelectricity.^14^ Unlike the simple polarity reversal of ferroelectrics, the polar structure is different from the original antipolar structure and can be regarded as a new electrically induced phase. Such phase transformations often result in unexpectedly large changes in the polarization, strain, and/or entropy, which has resulted in antiferroelectrics being reconsidered as promising materials for practical applications such as actuators, sensors, and energy and charge storage devices.^14^–^16^

**Chart 1:**
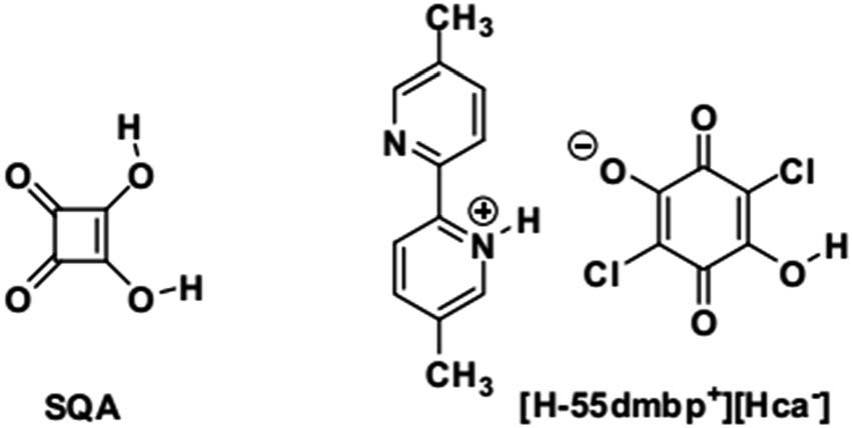


However, there are few examples^6^ and studies devoted to polarization switching in organic antiferroelectric crystals. Squaric acid (3,4-dihydroxy-3-cyclobutene-1,2-dione; SQA) is symbolic in that its antiferroelectricity has not been demonstrated directly by polarization switching despite more than half a century of research after its synthesis.^17^,^18^ Application of a strong electric field to an SQA crystal is expected to induce a polarization as strong as the spontaneous polarization of CRCA, considering the close chemical analogies of the oxocarbon acids.^19^ Here, we present characteristic *P*–*E* curves as firm evidence of the antiferroelectricity and demonstrate the microscopic pictures of observed polarization switching for two hydrogen-bonded compounds: a prototropic crystal of SQA and a supramolecular crystal of [H-55dmbp][Hca] salt^20^ (55dmbp = 5,5′-dimethyl-2,2′-bipyridine; H_2_ca = chloranilic acid) (Chart 1).

## Results and discussion

### Structural assessment

**Fig. 1:**
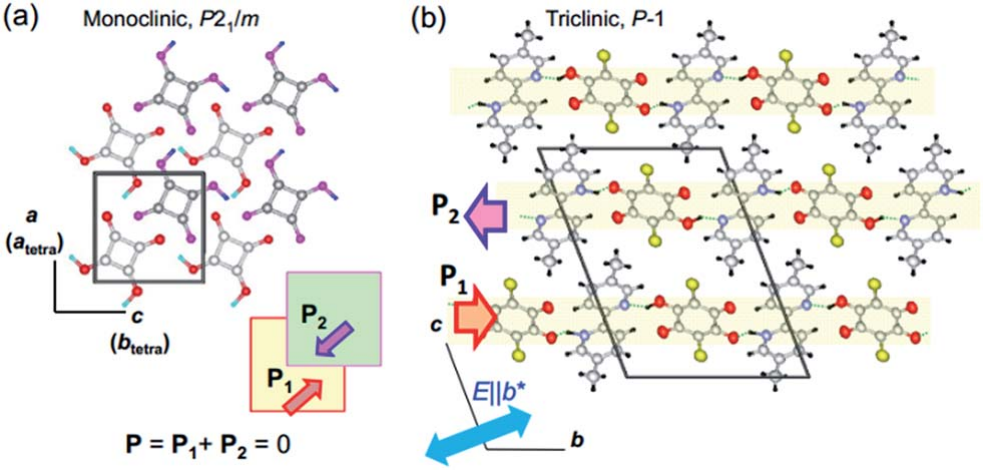
Molecular arrangements and directions of sublattice polarization in antipolar crystal structures determined at room temperature (redrawn after experimental results in ref. 20 and 21). (a) Crystal structure of SQA viewed along the crystallographic monoclinic *b* (pseudo-tetragonal *c*) axis. Thick arrows on the hydrogen bonded sheet (shaded squares) indicate the direction of the theoretically calculated sublattice polarizations **P**_1_ in the *y* = 1/4 layer and **P**_2_ in the *y* = 3/4 layer. (b) Crystallographic *a*-axis projection with the direction of the sublattice (chain) polarizations (thick arrows) in the [H-55dmbp][Hca] salt indicated.

One of the signatures of antiferroelectricity in the SQA and [H-55dmbp][Hca] crystals is the structural phase transition at high temperature (373 K and 318 K, respectively).^18^,^20^ The room-temperature crystal structures in Fig. 1 are based on the atomic coordinates determined in previous work^21^ and represent those of the low-temperature phase. The planar square-shaped molecules are hydrogen bonded in a flat sheet parallel to the crystal *ac* plane in the SQA crystal,^21^ whereas the acid and base molecules are alternately hydrogen bonded into a ribbon-like supramolecule parallel to the *b*-direction in the [H-55dmbp][Hca] crystal.^20^ All the protons within the O···O and O···N hydrogen bonds are ordered in the low-temperature phase and become disordered over two positions in the high-temperature phase according to neutron and X-ray diffraction studies.^20^,^22^ The phase transition, therefore, has some characteristics of order–disorder dynamics.

At high temperatures, the body-centered tetragonal lattice of space group *I*4/*m* comprises disordered protons and a C_4_O_4_^2–^ unit of square (*D*_4h_) symmetry, and each molecular sheet has no net dipoles.^22^ This symmetry is reduced to a primitive monoclinic lattice with space group *P*2_1_/*m* below *T*_c_. In the low-temperature phase, proton ordering is accompanied by *C*_1h_-type asymmetric distortion of the C_4_O_4_ core with clear alternating single and double bonds. Each molecular sheet acquires an electric polarization in a direction parallel to the *ac* plane, whereas its neighboring sheets related by inversion symmetry obtain the opposite polarization, thus constructing the antipolar crystal structure. The phase transition from nonpolar to antipolar structures manifests itself in the antiferroelectric ordering, and the former and later phases are referred to here as the paraelectric (PE) and antiferroelectric (AFE) phases, respectively. Since the crystallographic axes are defined differently in the two phases, the crystallographic directions are presented in the monoclinic (AFE form) setting unless noted by the subscript “tetra” (for instance, see Fig. 1a).

The proton ordering in the [H-55dmbp][Hca] crystal below *T*_c_ is accompanied by a simple unit-cell doubling of the triclinic cell as well as the emergence of a dipole moment in each supramolecular ribbon.^20^ The neighboring ribbon in the *c*-direction has the opposite polarization (arrows in Fig. 1b).

### Theoretical sublattice polarizations

**Fig. 2:**
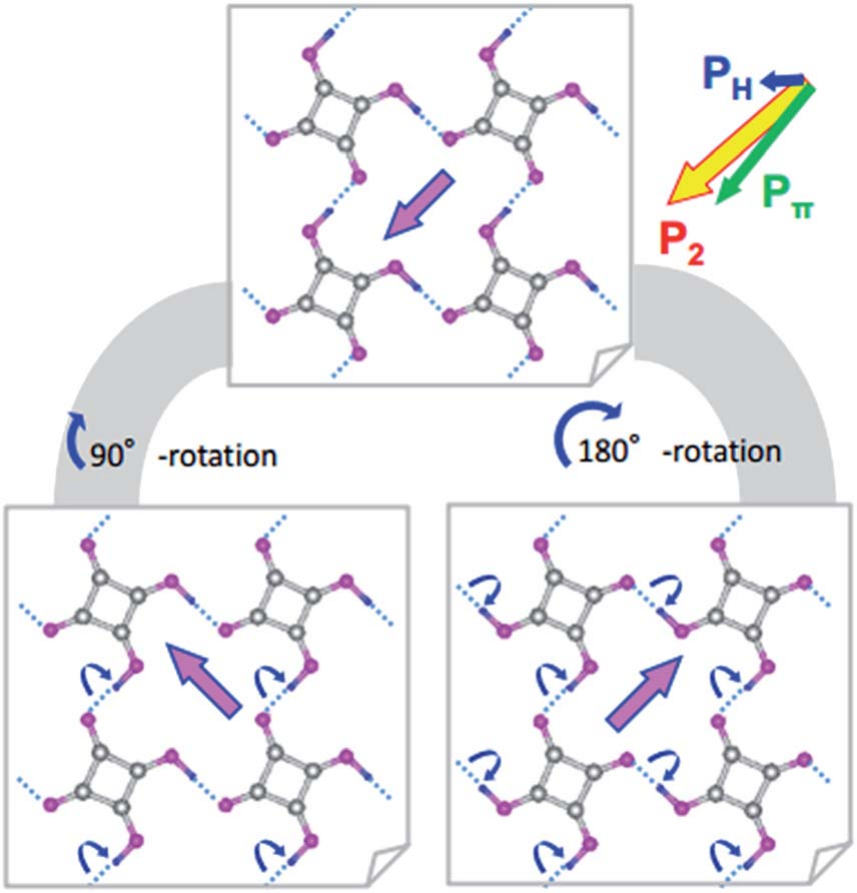
Microscopic origins of the sublattice polarization and rotations exemplified by the polarization vector **P**_2_ in the *y* = 3/4 layer in the SQA crystal. The theoretically calculated sublattice polarization **P**_2_ is divided into the ionic polarization **P**^H^ of displacing protons estimated under the point-charge approximation and the remainder contribution **P**^π^ mainly from the π-bond switching. Open arrows compare the directions and relative amplitudes. Blue dotted lines represent the intermolecular hydrogen bonds. The bottom two structures schematize the proton-transfer processes (round arrows) required for the 90° rotation (left) and 180° inversion (right) of polarization **P**_2_.

According to the early Landau-type model of Kittel,^11^ the antiferroelectric state is described by two interpenetrating sublattices with opposite polarizations. The two sublattices in the antipolar crystal structures correspond to two-dimensional sheets for SQA and one-dimensional ribbons for [H-55dmbp][Hca]. The sublattice polarizations **P**_1_ and **P**_2_ are defined by dividing the dipole moments of the sublattice by the unit cell volume *Ω*. Note that the directions of the thick open arrows in Fig. 1 represent those of the theoretically calculated **P**_1_ and **P**_2_ (= –**P**_1_). The sublattice polarizations **P**_1_ were computed using the Berry phase formalism^23^ (Fig. S1†) by employing the same procedure as that for ferroelectrics in our previous work except that the target polar crystal structure was constructed simply by removing sublattice 2 from the original AFE structure. The atomic positions at room temperature were taken from the neutron diffraction dataset^21^ for SQA and from the synchrotron X-ray diffraction dataset (CCDC1571765)^20^ for [H-55dmbp][Hca]. Only hydrogen atoms were computationally relaxed in the core locations so as to minimize the total energy, since the X-ray diffraction finds the corresponding electron density maxima with a large ambiguity in the X–H (X = C, O, or N) distances. Because the hydrogen positions were determined by neutron diffraction for SQA, a small computational adjustment of the O–H bond length from 1.034 to 1.052 Å resulted in little change in the calculated sheet polarization: (|**P**_1_|; *P*_1a_, *P*_1b_, *P*_1c*_) = (11.6; 7.6, 0, 8.8) μC cm^–2^ and (11.0; 7.3, 0, 8.2) μC cm^–2^ before and after relaxing the hydrogen atoms, respectively. A smaller sublattice (ribbon) polarization **P**_1_ was obtained for [H-55dmbp][Hca]; (|**P**_1_|; *P*_1a_, *P*_1b′_, *P*_1c*_) = (3.9; –1.2, 3.6, –0.1) μC cm^–2^ after optimization of the hydrogen positions with reasonable O–H and N–H bond lengths (1.028 and 1.090 Å, respectively). To evaluate the contributions from protons and switchable π-bond dipoles to the sublattice polarizations **P**_1_ in SQA, the point-charge model was applied to calculate the former contribution from protons displaced among molecular (anionic) cores,1
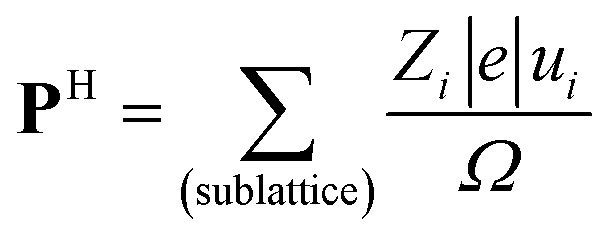
where *e* is the electron charge, *u*_*i*_ is the relative displacement of the static charges *Z*_*i*_ |*e*|, and *Z*_*i*_ is taken as +1 for protons and –2 for C_4_O_4_^2–^ molecular cores. Comparison of **P**^H^ with **P**_1_ revealed that a much larger **P**^π^ contribution, mainly from switchable π-bond dipoles, remains compared with that from **P**^H^: (|**P**|; *P*_a_, *P*_b_, *P*_c*_) = (2.8; 0.1, 0, 2.8) and (9.6; 7.5, 0, 6.0) μC cm^–2^ for **P**^H^ and **P**^π^, respectively. A very similar magnitude relationship has been reported for the spontaneous polarization of prototropic ferroelectrics.^7^ In the SQA crystal, the direction of sublattice polarization **P**_1_ is governed by that of **P**^π^ in the [101] direction rather than **P**^H^ in the [100] direction (see arrows in Fig. 2).

Switching from the antipolar to polar structure in the [H-55dmbp][Hca] crystal is more straightforward because the sublattice polarization is approximately parallel to the supramolecular ribbons and the one-dimensional character only allows its inversion. With the electric field applied, cooperative proton transfer takes place between the acid and base so as to invert the sublattice polarization from antiparallel to parallel to the external field. In contrast, the pseudo-tetragonal symmetry of the SQA crystal permits the additional 90° rotation of two-dimensional sublattice polarizations in four directions. Fig. 2 illustrates the microscopic structural changes with cooperative proton transfer for polarization rotations. Note that the 90° rotation requires transfer of only one proton per molecule, whereas the 180° inversion involves double proton transfer, which is also the case in the ferroelectric CRCA crystal. In the simplest picture, the 180° inversion of the sublattice polarizations (if assumed for SQA) could produce the optimum polarization of 2|**P**_1_| as the field-induced response. The theoretically estimated 2|**P**_1_| of SQA is 23.2 μC cm^–2^, which agrees well with the calculated polarization (23.3 μC cm^–2^) reported by Ishii *et al.*^24^ and expectedly approaches the record of spontaneous polarization of an organic ferroelectric (30 μC cm^–2^ of CRCA). It is noted that the 2|**P**_1_| = 7.8 μC cm^–2^ and *T*_c_ = 318 K of antiferroelectric [H-55dmbp][Hca] obey the same structure–property relationship as that observed for supramolecular ferroelectrics (Fig. S2†). The findings of the theoretical calculations imply that hydrogen-bonded organic ferroelectrics and antiferroelectrics have many common design principles for better polarization performance and thermal stability.

### Dielectric properties

**Fig. 3:**
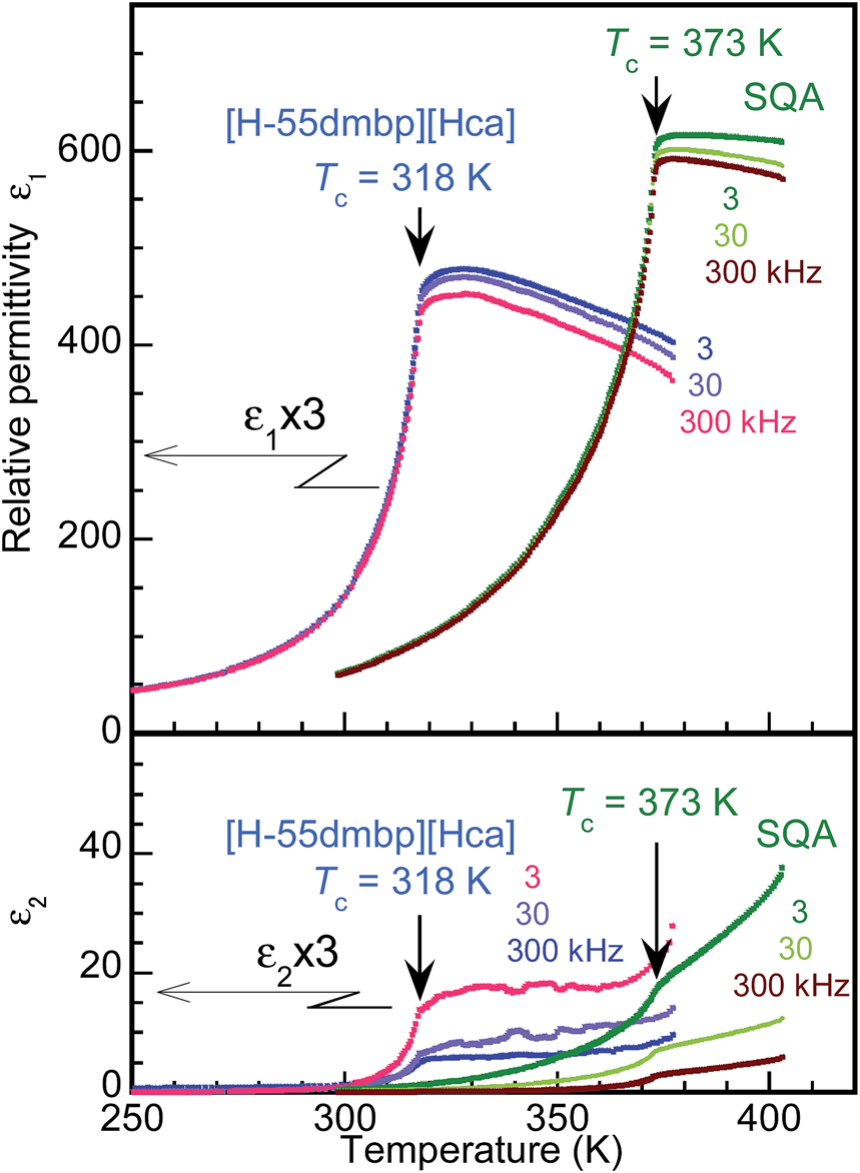
Temperature variations of the real (*ε*_1_, top) and imaginary parts (*ε*_2_, bottom) of the relative dielectric constant for an SQA crystal in the *E*||[100]_tetra_ configuration and a [H-55dmbp][Hca] crystal in the *E*||b* configuration at frequencies of 3, 30, and 300 Hz. The dielectric responses of [H-55dmbp][Hca] are scaled by a factor of 3 for clarity.

The dielectric properties of the SQA and [H-55dmbp][Hca] crystals were found to have a similar temperature dependence as shown by the real and imaginary parts (*ε*_1_ and *ε*_2_) of the dielectric constant at three different frequencies in Fig. 3. The ac electric field was applied along [100]_tetra_ in the tetragonal setting for the SQA crystal without distinction of the *a* and *c* axes, which is difficult from the crystal appearance. There are several precedent studies on the permittivity at ambient and high pressures for SQA.^25^–^31^ This investigation examined the dielectric properties after the *P*–*E* hysteresis experiments on the same crystal specimen, because the maximum permittivity ranging from 300 to 620 was reported to depend on the degree of crystal twinning. Data for [H-55dmbp][Hca] were previously collected by applying the electric field normal to the (010) plane and partly reported in ref. 20.

The phase transition of SQA was confirmed by the onset of the steep drop in the permittivity below *T*_c_ = 373 K, which is very similar to the temperature dependence of [H-55dmbp][Hca] below *T*_c_ = 318 K. The maximum permittivity of 620 is comparable to the optimum values observed in previous work,^18^,^27^ and the least effect of averaging the *a*- and *c*-direction permittivities implies a single or nearly single domain state for the used specimen.

For both compounds, the dissipation factor (tan *δ* = *ε*_2_/*ε*_1_) is very low (less than 0.01) around room temperature and gradually increases with temperature until it saturates still at a low level (<0.05) for temperatures beyond *T*_c_. These features are suitable for low-energy-loss storage.

### Field-induced phase transitions

In the *P*–*E* hysteresis experiments, the configurations between the crystal and applied electric field were the same as those employed for the permittivity measurements above. The SQA and [H-55dmbp][Hca] crystals behave as simple dielectrics exhibiting a linear *P*–*E* relationship unless the field amplitude was increased beyond 100 kV cm^–1^. This would be a plausible reason why observation of antiferroelectric switching in SQA has been elusive.

**Fig. 4:**
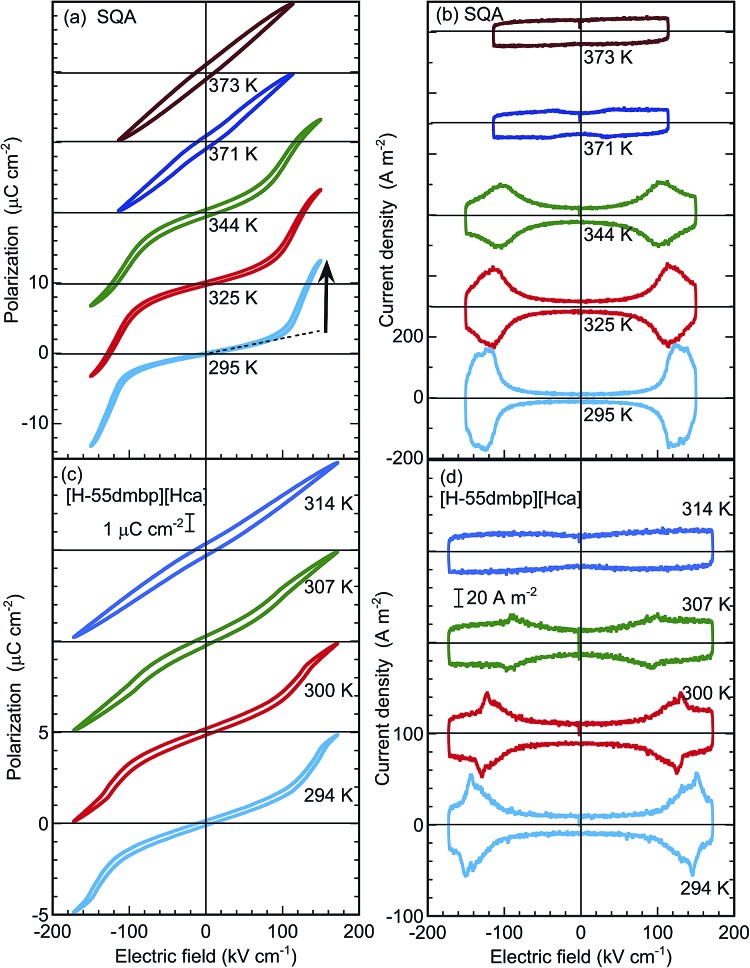
Antiferroelectric switching at various temperatures. Electric polarization (*P*) *versus* electric field (*E*) hysteresis loops at *f* = 100 Hz (a, c) and corresponding current density (*J*) *versus E* (b, d) of [100]_tetra_ polarization in SQA and b*-direction polarization in [H-55dmbp][Hca] salt.

The satisfactory collection of double hysteresis curves required a maximum field amplitude of 150–170 kV cm^–1^ for both compounds at room temperature (Fig. 4a and c). A steep increase/decrease in the polarization in the high-field region is accompanied by a displacement current, which exhibited two pairs of peaks in the corresponding current density (*J*) *versus* electric field (*E*) curve (Fig. 4b and d). These features are definitive of antiferroelectricity. At room temperature, the field-induced maximum polarization *P*_m_ of SQA is about 13.3 μC cm^–2^ accompanied by a jump Δ*P* of ∼10.5 μC cm^–2^ (arrow in Fig. 4a), and these values are comparable in magnitude to the sheet polarization calculated above. The switchable polarization of SQA is larger than those of existing organic antiferroelectrics such as benzimidazoles^6^ (9–10 μC cm^–2^, see Table 1). The [H-55dmbp][Hca] crystal exhibited a smaller *P*_m_ of ∼5 μC cm^–2^ and a Δ*P* of ∼3 μC cm^–2^. With increasing temperature, the *P*–*E* curves smear out the polarization jump until the linear *P*–*E* relationship for normal paraelectrics survives at *T*_c_. This behavior corresponds to the collapse of peaks by broadening in the *J*–*E* curves. Note that each polarization loop was always very slim, indicating minimal hysteresis.

**Table 1:** Antiferroelectric and energy storage properties of hydrogen-bonded organic crystals at room temperature

Compound^*a*^	AFE-to-PE transition temp.	Polarization	Switching field	Stored energy	Efficiency	Hysteresis conditions^*b*^		
	*T*_c_ (K)	*P*_m_ (Δ*P*) (μC cm^–2^)	*E*_sw_ (Δ*E*_sw_) (kV cm^–1^)	*U*_s_ (*U*_r_) (J cm^–3^)	*η*	*f* (Hz)	*E*_m_ (kV cm^–1^)	**E**-direction
SQA	373	13.3 (10.5)	124 (4.6)	1.53 (1.44)	0.94	100	151	||[100]_tetra_
[H-55dmbp][Hca]	318	4.9 (2.7)	148 (5.5)	0.564 (0.510)	0.90	100	173	||b*
TFMBI^*c*^	>393	9.0 (7.8)	12.9 (7.7)	0.137 (0.061)	0.44	0.2	22	||[001]
DFMBI^*c*^	>413	10.5 (8.0)	67.2 (13.3)	0.673 (0.528)	0.78	2	86	||[001]
TCMBI^*c*^	>413	9.4 (7.0)	49.4 (24.0)	0.538 (0.333)	0.62	10	81	||[100]

^*a*^TFMBI = 2-trifluoromethylbenzimidazole, DFMBI = 2-difluoromethylbenzimidazole, TCMBI = 2-trichloromethylbenzimidazole.

^*b*^ *f* = applied frequency of triangular waves, *E*_m_ = maximum field amplitude applied.

^*c*^The analysis employed the data in ref. 6 for three benzimidazoles.

**Fig. 5:**
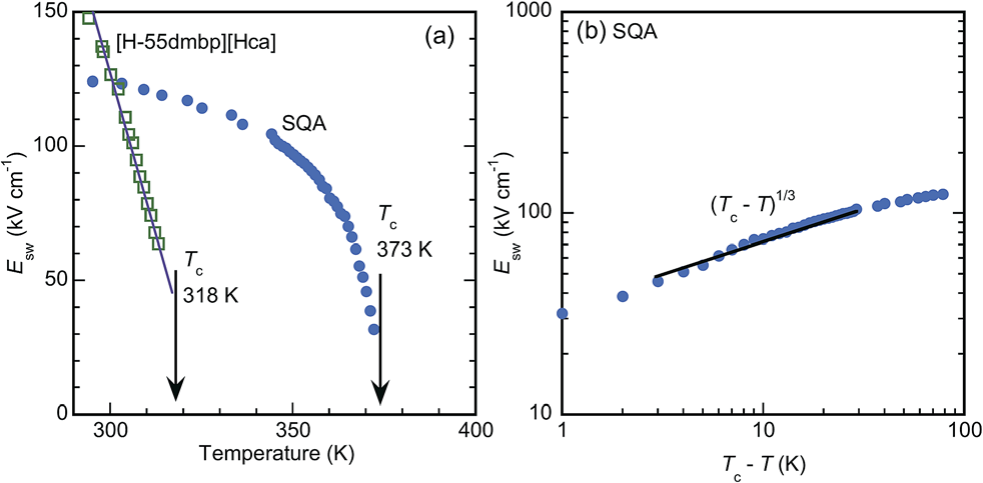
Temperature variation of the antiferroelectric switching field. (a) Linear plot for [100]_tetra_ polarization in SQA and b*-direction polarization in [H-55dmbp][Hca] salt. Arrows indicate the antiferroelectric phase-transition temperatures. (b) Logarithmic plot for [100]_tetra_ polarization in SQA.

The critical (switching) field *E*_sw_ for the field-induced phase transition was estimated by averaging the field amplitudes over the four peak positions. As shown by the temperature (*T*) dependence in Fig. 5a, *E*_sw_ rapidly approaches zero as *T* approaches *T*_c_. This observation manifests the antiferroelectric phase transition. The *E*_sw_*versus T* curves correspond to the phase boundary between the antiferroelectric and field-induced ferroelectric phases in the *E*–*T* diagram. The [H-55dmbp][Hca] crystal exhibited a linear temperature dependence on *E*_sw_, which is often the case for antiferroelectric oxides. On the other hand, the *E*_sw_ of SQA revealed unusual critical behavior; *E*_sw_ ∼ (*T*_c_ – *T*)^1/*Φ*^ with *Φ* ∼ 3 (Fig. 5b). A change in the critical behavior was also noted by the change in slope at *T*_c_ – *T* ∼ 10 K. Earlier work discovered a marked change in the critical index for specific heat at this temperature and attributed it to the crossover from two-dimensional to three-dimensional characters.^18^,^27^,^32^ Although a full analysis is beyond the scope of this study, the distinct critical behaviors of the two compounds as well as the crossover suggest the unique physical aspects of two-dimensional ferroelectricity in SQA.

### Energy storage properties

The *P*–*E* hysteresis curve of SQA reveals some advantageous characteristics for energy storage applications. In general, dielectrics can store an electric energy density *U*_s_ and recover an energy density *U*_r_ with an efficiency of *η* as a capacitor according to the relations,2
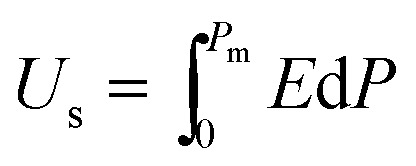

3
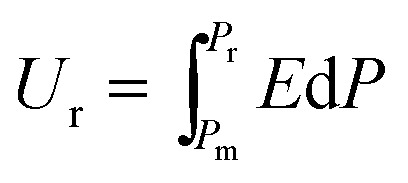

**Fig. 6:**
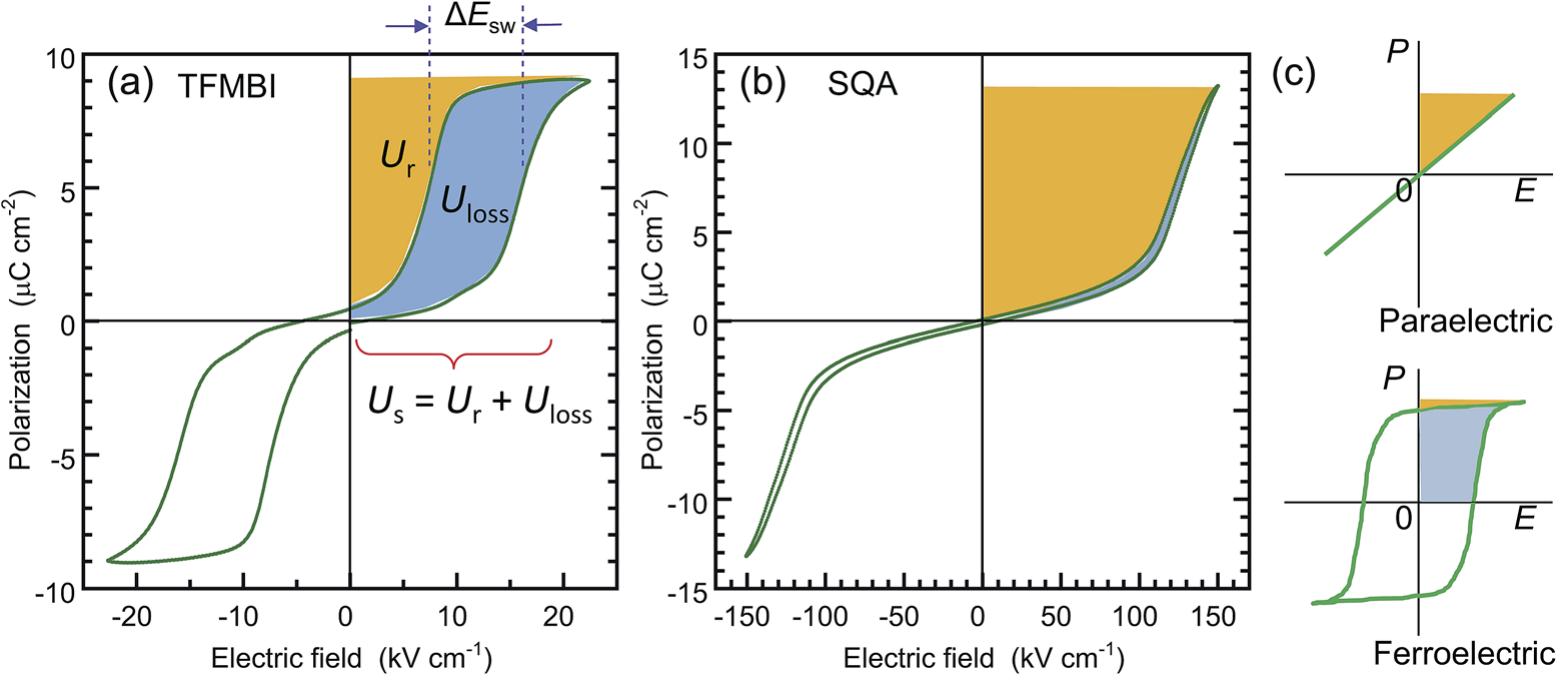
Comparison of the recoverable energy density *U*_r_ (orange area) and unrecoverable energy density *U*_loss_ (blue area) from the viewpoint of energy storage applications. (a) Antiferroelectric 2-trifluoromethybenzimidazole (TFMBI) crystal with small energy storage and low efficiency. The *P*–*E* curve was redrawn after the previously reported data^6^ collected at a frequency of 0.2 Hz. (b) Antiferroelectric SQA crystal with large energy storage and excellent efficiency. (c) Schematic illustrations of the energy storage properties in the linear dielectric and ferroelectric.

4
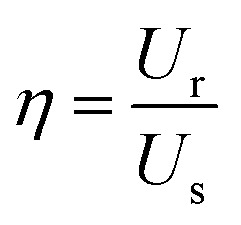
where *P*_m_ and *P*_r_ are the maximum polarization and remanent polarization, respectively.^1^,^14^,^15^,^33^,^34^ These energy densities can be obtained from the numerical integration of the charging and discharging *P*–*E* curves. As exemplified in Fig. 6a by our previously reported data of the antiferroelectric 2-trifluoromethybenzimidazole (TFMBI) crystal,^6^ most antiferroelectrics exhibit substantial hysteresis so that *U*_s_ contains a large unrecoverable component *U*_loss_ (blue area) in addition to the recoverable *U*_r_ (orange area). It becomes evident from the schematic illustration in Fig. 6 that antiferroelectrics having a sufficiently large *P*_m_, small *P*_r_ (∼0), and high *E*_sw_ can store a large *U*_s_ much more effectively than ferroelectrics and linear paraelectrics. One of the critical requisites to be settled especially for high-power capacitors is a high efficiency *η* close to unity, which necessitates small hysteresis between the forward and backward switching.^14^,^15^,^33^,^34^

Table 1 summarizes the antiferroelectric properties and energy storage performances obtained from the double hysteresis loops. For comparison, we also analyzed the previous data on three benzimidazoles.^6^ Here, the hysteresis width Δ*E*_sw_ was defined by the difference in the peak field between the charging and discharging *J*–*E* curves. A low efficiency, *η* = 0.44, was found for TFMBI exhibiting clear hysteresis (Fig. 6a). In sharp contrast, the SQA crystal exhibits a *P*–*E* hysteresis curve ideal for energy-storage properties; a much larger *P*_m_ and higher *E*_sw_ exceeding 120 kV cm^–1^ at room temperature give rise to a much larger *U*_s_ of 1.53 J cm^–3^ and *U*_r_ of 1.44 J cm^–3^ under the maximum field amplitude *E*_m_ = 151 kV cm^–1^ and very slim hysteresis of only 4.6 kV cm^–1^ gives rise to an excellent efficiency of *η* = 0.94 (Fig. 6b). Both the energy-storage density and efficiency are in the highest level among those of recently reported lead-free antiferroelectrics (for instance see ref. 35 for bulk antiferroelectrics). A high efficiency of *η* = 0.90 was similarly deduced from the slim *P*–*E* loop (Δ*E*_sw_ = 5.5 kV cm^–1^) of the [H-55dmbp][Hca] crystal. The origin of the high efficiency can be attributed to an elevated *E*_sw_ without an increase in Δ*E*_sw_ in comparison with benzimidazoles.

### Field-induced structures of SQA crystal

**Fig. 7:**
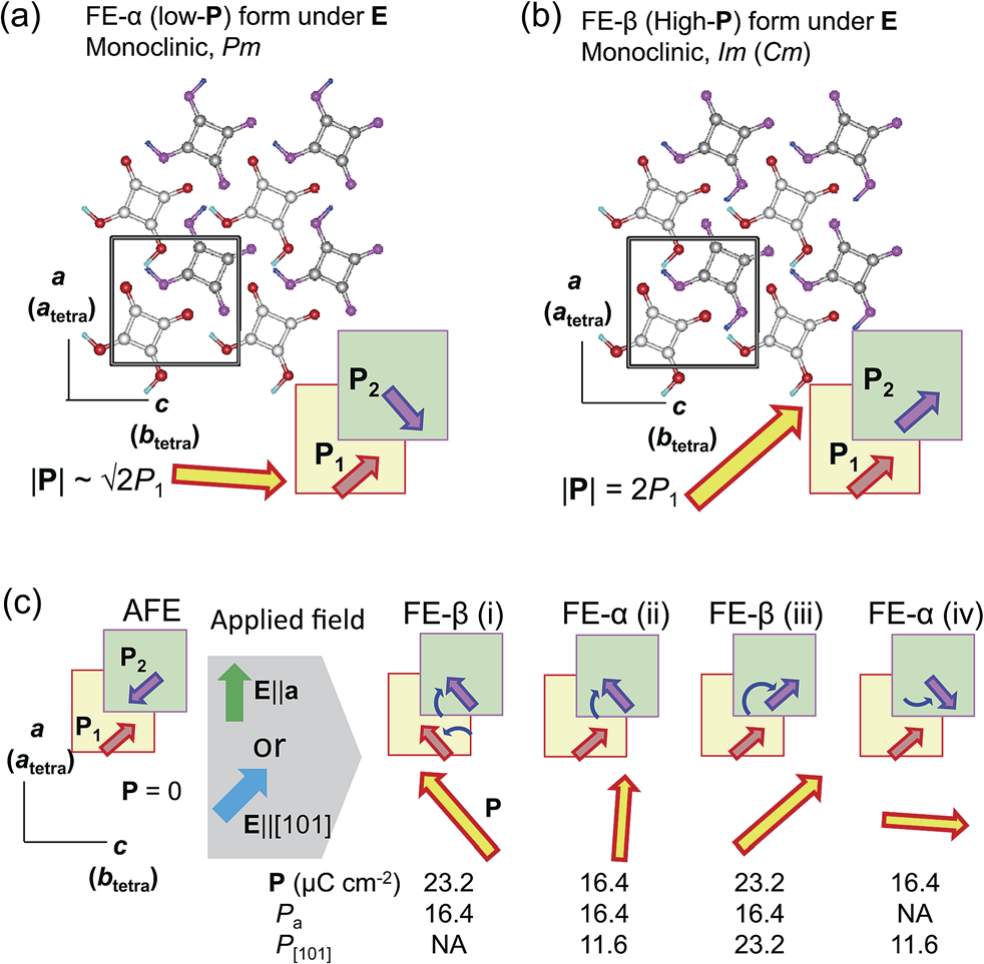
Field-induced hypothetical crystal structures viewed along the crystallographic monoclinic *b* (pseudo-tetragonal *c*) axis of SQA. (a) Field-induced low-polarization (FE-α) and (b) high-polarization (FE-β) structures. Thick arrows on the hydrogen-bonded sheet (shaded squares) indicate the directions of the sublattice polarizations **P**_1_ in the *y* = 1/4 layer and **P**_2_ in the *y* = 3/4 layer as well as that of the total **P** = **P**_1_ + **P**_2_. (c) Candidate polarization states under an applied electric field with a *E*||*a* or *E*||[101] configuration. Round arrows indicate the rotation of the sublattice polarizations during the switching from the AFE to FE structure; *P*_a_ and *P*_[101]_ denote the respective field-direction components of induced total polarization **P**.

The discovery of the double *P*–*E* hysteresis in the two-dimensional SQA crystal addresses another unique possibility in electric-field-induced structural changes. Based on the sublattice model depicted in Fig. 2a, 90° rotation and 180° inversion of the sublattice sheet polarization **P**_2_ transform the AFE structure into two kinds of FE structures of different total polarization. In the former ferroelectric structure, called FE-α hereafter (Fig. 7a), the crossed **P**_1_ and **P**_2_ yield a **P** of lower magnitude √2*P*_1_ (=16.4 μC cm^–2^) directed along [100]_tetra_. The latter FE-β structure (Fig. 7b) of the aligned sublattice polarizations has the largest **P** (magnitude 2*P*_1_ = 23.2 μC cm^–2^) directed along [110]_tetra_. These polar structures retain mirror symmetry, but the lattice symmetry is lowered to a monoclinic lattice, which is of a primitive type for FE-α and of a *C*-centered type for FE-β. The three structures, AFE, FE-α, and FE-β, differ in terms of crystal symmetry and should be allocated to distinct crystal phases.

With these two FE structures in mind, Fig. 7c illustrates the possible structural changes with 90° rotation and/or 180° inversion of the sublattice polarizations under the two different applied field **E** configurations. The **E**||[100] configuration is representative of the above experimental observations. The three configurations FE-β(i), FE-α(ii), and FE-β(iii) allow for an energetically attractive interaction between the induced polarization **P** and **E**||[100], but they happen to exhibit the same field-direction components (*P*_a_ = 16.4 μC cm^–2^) and can explain the experimentally field-induced polarization of about 14 μC cm^–2^. On the other hand, the above *P*–*E* hysteresis results alone cannot identify the actual field-induced structure. We therefore examined the reference experiments with **E**||[110]_tetra_. Among the three energetically attractive configurations with **E**||[101], FE-β(iii) exhibits an optimum field-direction component (*P*_[101]_), which is twice those for FE-α(ii) and FE-α(iv). Compared with the **E**||[100]_tetra_ dataset, the hysteresis loop with **E**||[110]_tetra_ exhibited much smaller polarizations and a higher switching field (Fig. S3†). The reduced polarization with the **E**||[110]_tetra_ configuration suggests the FE-α structure. Furthermore, the increased switching field manifests itself in that the direction of induced polarization **P** is close to [100]_tetra_ rather than [110]_tetra_. In summary, the FE-α phase is induced as the new third crystal form with energetically more stable structure rather than FE-β. This observation leaves the novel possibility of successive switching to the FE-β phase (undiscovered forth crystal form) with a much stronger field **E**||[110]_tetra_, although trials with the higher field have so far failed because of dielectric breakdown.

## Conclusions

We have demonstrated direct evidence of antiferroelectricity by applying a strong electric field to the prototropic SQA crystal and the supramolecular [H-55dmbp][Hca] crystal. The observations of double hysteresis loops manifest the field-induced switching of polarizations on sublattices of hydrogen-bonded two-dimensional sheets or one-dimensional ribbons. The field-induced polarization of SQA is quite large and reasonably explained by the theoretical calculation of the sheet polarization. The pseudo-tetragonal lattice of SQA permits unique switching topologies producing two different ferroelectric phases FE-α and FE-β of low and high polarizations, respectively. By tilting the applied field direction, the electrically induced phase is identified as FE-α switched through a 90° rotation of the sublattice polarization. From the viewpoint of applications, the strongly induced polarization, high switching field, and quite slim hysteresis observed in the *P*–*E* curve for SQA are just the advantages needed for high-efficiency energy storage devices.

## Experimental section

### Preparation

Repeated recrystallization of purchased SQA from hot aqueous solution gave colorless bipyramid crystals of about 0.8–1 mm in size. Slow evaporation of the aqueous solution of purified SQA in a Teflon beaker in the dark gave larger pyramidal or bipyramid crystals of 3 mm in size (Fig. S4†). Dark purplish brown plate crystals of [H-55dmbp][Hca] were grown by diffusing the acid (H_2_ca) and base (55dmbp) in acetone after the two component compounds were separately purified by repeated vacuum sublimations.

### Electric measurements

All electric measurements were obtained from single crystals with painted silver electrodes. For the SQA crystals, the top and bottom edges of the bipyramid were easily cleaved with a blade along the (001)_tetra_ plane, which is parallel to the bottom plane of the pyramid (Fig. S4b†). The resultant plates were then cut along the (100)_tetra_ plane (or (110)_tetra_ plane) at both ends for flat electrodes. As-grown crystals were used for [H-55dmbp][Hca]. The *P*–*E* hysteresis curves were collected on a ferroelectrics evaluation system (Toyo Corporation, FCE-1) consisting of a current/charge–voltage converter (model 6252), an arbitrary waveform generator (Biomation 2414B), an analog-to-digital converter (WaveBook 516), and a voltage amplifier (NF Corporation, HVA4321). The measurements at room temperature were performed with a high-voltage triangular wave field and various alternating frequencies. The crystals were immersed in silicon oil to prevent atmospheric discharge under a high electric field (>30 kV cm^–1^). After the *P*–*E* hysteresis experiments, the dielectric constant was measured on the same SQA crystal specimen with an LCR meter (HP 4284A).

### Theoretical calculations

Electronic polarizations were evaluated using the Berry phase approach^23^,^36^ with the QMAS code^37^ based on the projector augmented-wave method^38^ and the plane-wave basis set. To describe the electronic exchange–correlation energy, the Perdew–Burke–Ernzerhof (PBE) version of the generalized gradient approximation (GGA)^39^ was used. The total polarization is obtained as the sum of the electronic polarization and ionic polarization. Further details are described elsewhere.^40^,^41^ The target ferroelectric structures (degree of polar distortion *λ* = 1) were constructed from the atomic coordinates of all the non-hydrogen atoms determined by the experiments. The locations of the hydrogen atoms were computationally relaxed so as to minimize the total energy. The reference paraelectric structures (*λ* = 0) were constructed from averaged molecular structures for *λ* = ±1.

## Conflicts of interest

There are no conflicts to declare.

## Supplementary Material

Supplementary information
