# Supplementary material for: Strong polarization switching with low-energy loss in hydrogen-bonded organic antiferroelectrics
Source: Chem Sci. 2017 Nov 1;9(2):425–32. doi: 10.1039/c7sc03859c (PMC5872138; doi:10.1039/c7sc03859c)
Supplement: Supplementary file 1 [file SC-009-C7SC03859C-s001.pdf]

Electronic Supplementary Material (ESI) for Chemical Science.
This journal is © The Royal Society of Chemistry 2017

Strong Polarization Switching with Low-Energy Loss in Hydrogen-Bonded Organic Antiferroelectrics

Sachio Horiuchi,[a] Reiji Kumai[b] and Shoji Ishibashi[c]

[a]Flexible Electronics Research Center (FLEC), National Institute of Advanced Industrial Science and Technology (AIST), Tsukuba, Ibaraki 305-8565, Japan

[b]Condensed Matter Research Center (CMRC) and Photon Factory, Institute of Materials Structure Science, High Energy Accelerator Research Organization (KEK), Tsukuba, Ibaraki 305-0801, Japan

[c]Research Center for Computational Design of Advanced Functional Materials (CD-FMat), National Institute of Advanced Industrial Science and Technology (AIST), Tsukuba, Ibaraki 305-8568, Japan

Correspondence and requests for materials should be addressed to S.H. (e-mail: s-horiuchi@aist.go.jp)

Electronic Supplementary Information

Experimental details

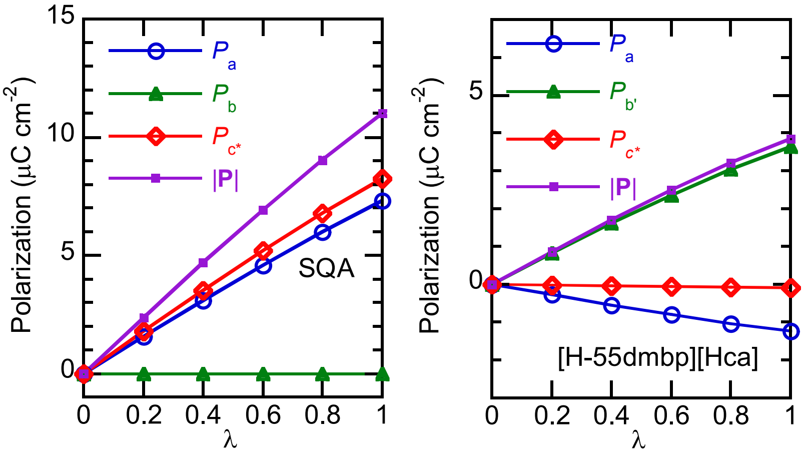

Figure S1. Evolution of the sublattice polarization as a function of the degree of polar distortion λ changing from the centrosymmetric reference (hypothetical paraelectric, λ = 0) to the fully polarized (ferroelectric, λ = 1) configuration. (a) Sheet polarization of the squaric acid (SQA) crystal and (b) ribbon polarization of the [H-55dmbp][Hca] crystal. In the λ = 1 structures of both crystals, the hydrogen positions were computationally relaxed. The polarization components were calculated using the Cartesian coordinate system; the *b*′-direction is taken as being perpendicular to the crystallographic *a* and *c** axes for the triclinic crystal of [H55dmbp][Hca].

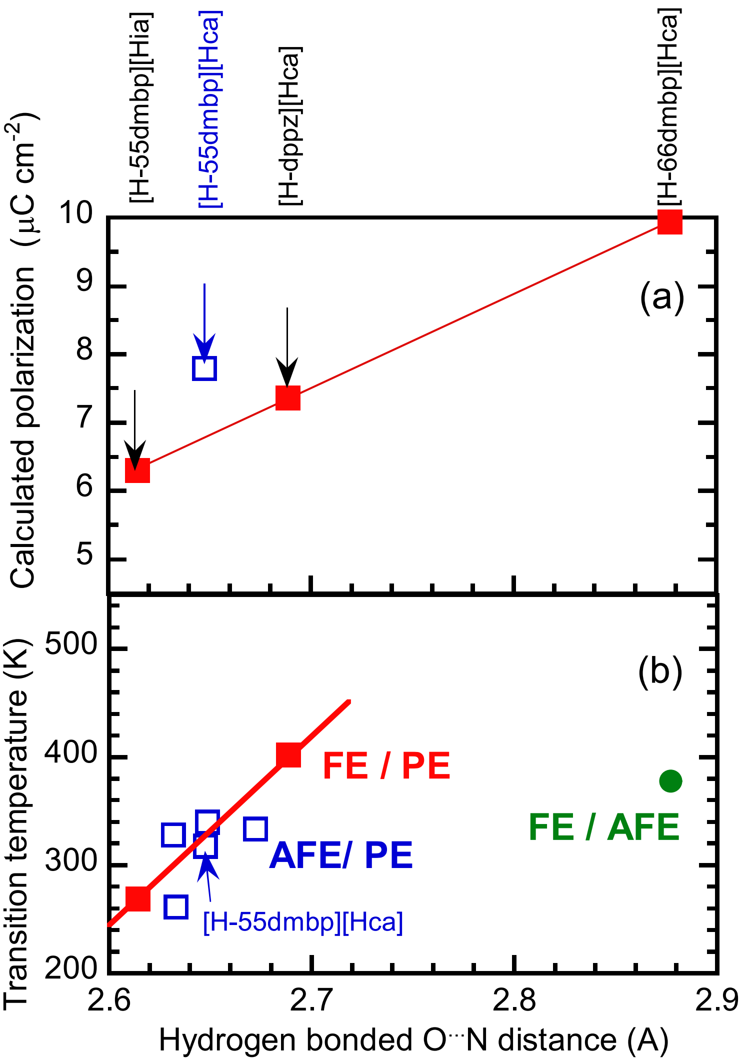

Figure S2. Comparison of the properties between the antiferroelectric [H-55dmbp][Hca] and ferroelectric acid/base supramolecular crystals of anilic acids as a function of the hydrogen-bonded O···N distance. (a) Induced polarization 2|$\mathbf{P}_1$| in the sublattice model (blue open squares) in comparison with the theoretical spontaneous polarization of the ferroelectric compounds (red filled squares). (b) Phase-transition temperature. Orange filled and open diamonds represent the ferroelectric and antiferroelectric transition (Curie) temperature T_c, respectively. The green circle indicates the ferroelectric-to-antiferroelectric phase-transition point of α-[H-66dmbp][Hca]. H_2ia = iodanilic acid, dppz = 2,3-di(2-pyridinyl)pyrazine, 66dmbp = 6,6'-dimethyl-2,2'-bipyridine. For a systematic comparison, the plot includes our previous data reported in ref. 1.

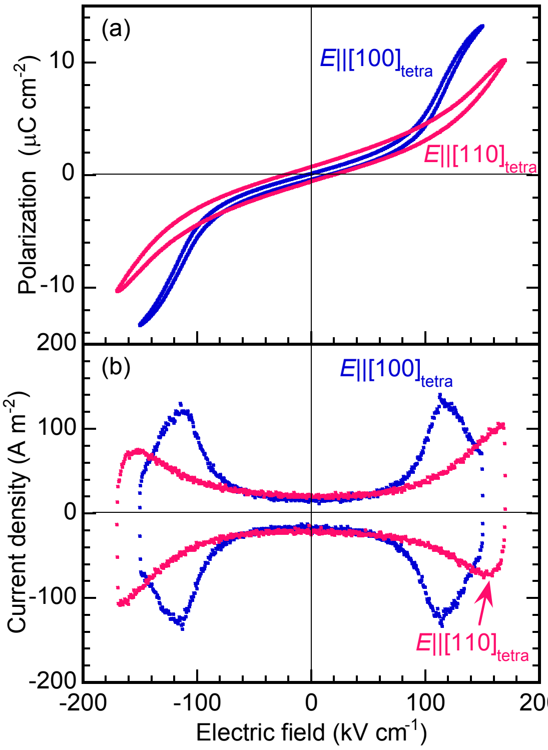

Figure S3. Antiferroelectric switching with **E**||[100]$_{tetra}$ and **E**||[110]$_{tetra}$ configurations in SQA crystal at T = 324 K and f = 100 Hz. (a) P–E hysteresis loops. (b) Corresponding J–E curves.

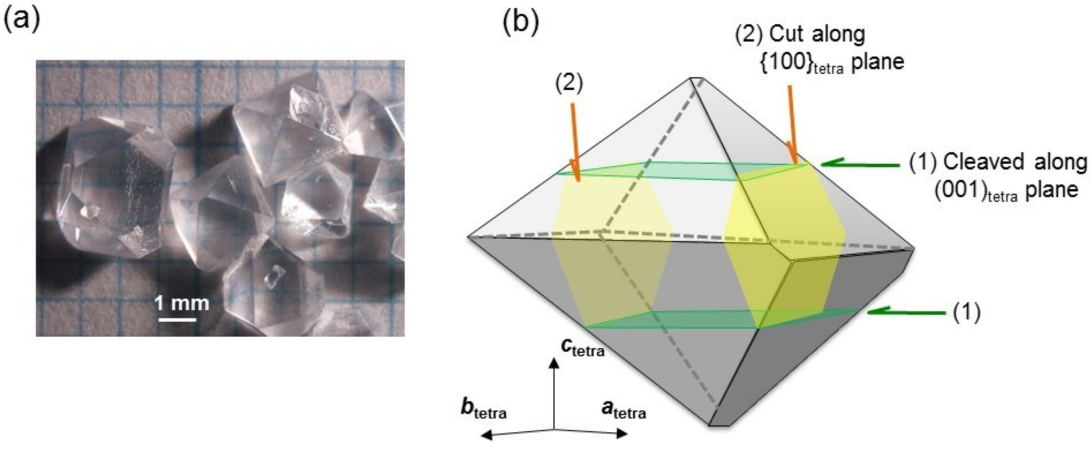

Figure S4. Squaric acid. (a) Photograph of crystals. (b) Schematic of the crystal cutting for the electric measurements.

1 K. Kobayashi, S. Horiuchi, S. Ishibashi, F. Kagawa, Y. Murakami and R. Kumai, *Chem. –Eur. J.*, 2014, **20**, 17515.
